# RANK Signaling in the Differentiation and Regeneration of Thymic Epithelial Cells

**DOI:** 10.3389/fimmu.2020.623265

**Published:** 2021-01-22

**Authors:** Magali Irla

**Affiliations:** Aix Marseille Univ, CNRS, INSERM, CIML, Centre d’Immunologie de Marseille-Luminy, Marseille, France

**Keywords:** bone marrow transplantation, central tolerance, receptor activator of nuclear factor kappa-B, thymic crosstalk, thymic epithelial cells, thymic regeneration

## Abstract

Thymic epithelial cells (TECs) provide essential clues for the proliferation, survival, migration, and differentiation of thymocytes. Recent advances in mouse and human have revealed that TECs constitute a highly heterogeneous cell population with distinct functional properties. Importantly, TECs are sensitive to thymic damages engendered by myeloablative conditioning regimen used for bone marrow transplantation. These detrimental effects on TECs delay *de novo* T-cell production, which can increase the risk of morbidity and mortality in many patients. Alike that TECs guide the development of thymocytes, reciprocally thymocytes control the differentiation and organization of TECs. These bidirectional interactions are referred to as thymic crosstalk. The tumor necrosis factor receptor superfamily (TNFRSF) member, receptor activator of nuclear factor kappa-B (RANK) and its cognate ligand RANKL have emerged as key players of the crosstalk between TECs and thymocytes. RANKL, mainly provided by positively selected CD4^+^ thymocytes and a subset of group 3 innate lymphoid cells, controls mTEC proliferation/differentiation and TEC regeneration. In this review, I discuss recent advances that have unraveled the high heterogeneity of TECs and the implication of the RANK-RANKL signaling axis in TEC differentiation and regeneration. Targeting this cell-signaling pathway opens novel therapeutic perspectives to recover TEC function and T-cell production.

## Introduction

The thymus supports the generation of distinct T-cell subsets such as conventional CD4^+^ and CD8^+^ T cells, Foxp3^+^ regulatory T cells, γδ T cells, and invariant natural killer T cells (iNKT). The development of these different T-cell subsets depends on stromal niches composed of thymic epithelial cells (TECs). TECs control T-cell development from the entry of T-cell progenitors to the egress of mature T cells. According to their anatomical localization and functional properties, TECs are subdivided into two main populations: cortical TECs (cTECs) and medullary TECs (mTECs). cTECs support the initial stages of T-cell development, including T-cell progenitor homing, T-cell lineage commitment, the expansion of immature thymocytes, death by neglect of thymocytes that do not recognize peptide-MHC complexes and positive selection of thymocytes into CD4^+^ and CD8^+^ T cells. By contrast, mTECs control late stages of T-cell development, mainly the induction of self-tolerance characterized by the clonal deletion of autoreactive thymocytes and CD4^+^ thymocyte diversion into the Foxp3^+^ regulatory T-cell lineage. Conversely, thymocytes control TEC expansion and differentiation. These bidirectional interactions between thymocytes and TECs are termed thymic crosstalk (1–3).

## TEC Heterogeneity in Mouse and Human

Historically, cTECs and mTECs were identified by histology using distinct markers such as cytokeratin 8 for cTECs and cytokeratin-5 and -14 for mTECs (4). TEC identification by flow cytometry on enzymatically-disaggregated thymus has greatly aided in studying TEC heterogeneity and functionality. TECs are non-hematopoietic cells, which express the Epithelial Cell Adhesion Molecule (EpCAM), and are generally identified as CD45^-^EpCAM1^+^. TECs can be further segregated into cTECs and mTECs based on the detection of Ly51 and reactivity to the lectin Ulex Europaeus Agglutinin 1 (UEA-1), respectively. cTECs and mTECs have distinct phenotypic and functional properties. Recent advances based on single-cell transcriptomic analyses have highlighted that TECs constitute a more diverse and dynamic population than previously thought.

## Features of Cortical TECs

cTECs express several molecules that govern the initial stages of T-cell development. They express CXCL12 and CCL25 chemokines that guide the homing of T-cell progenitors into the thymus (5, 6). cTECs also express the NOTCH ligand Delta-like 4 (DLL4), which induces the engagement of progenitors into the T-cell lineage (7, 8). Moreover, they express IL-7 and stem cell factor (SCF) cytokines that promote the survival and proliferation of immature thymocytes (9). They are equipped with protein degradation machineries important for the positive selection of CD4^+^ thymocytes such as the lysosomal endopeptidase cathepsin L (encoded by *Ctsl*) and the thymus-specific serine protease TSSP (encoded by *Prss16*) that contributes to MHC class II-associated self-peptide generation (10). They also express the thymoproteasome subunit β5t (encoded by *Psmb11*), which produces MHC class I-associated self-peptides required for the positive selection of CD8^+^ thymocytes (11).

cTECs are heterogeneous based on the expression level of MHCII, CD40, DLL4, and IL-7. Intriguingly, a cTEC subset specific of the perinatal thymus termed perinatal cTECs has been identified by single-cell transcriptomics (12). These cells, representing one-third of all TECs at 1 week of age, are highly proliferative and express synaptogyrin 1 (*Syngr1*) and G protein-coupled estrogen receptor 1 (*Gper1*) in addition to classical cTEC markers. Furthermore, by enveloping many viable double-positive (DP) thymocytes, a fraction of cTECs can form multi-cellular complexes called thymic nurse cells (TNCs) (13). TNCs likely provide a microenvironment favorable to secondary TCRα rearrangements in long-lived DP thymocytes, thereby optimizing TCR repertoire selection (14). Although TNCs remain poorly characterized, they exhibit a distinct gene expression profile characterized by high expression of CXCL12 and TSSP. TNCs thus constitute a cTEC subpopulation with distinct morphological and functional properties. Given that cTECs ensure multiple functions such as i) lymphoid progenitor homing, ii) T-cell lineage commitment, iii) immature thymocyte expansion, and iv) positive selection of thymocytes, it is likely that cTECs contain discrete functional subsets. Further investigations are required to clarify cTEC heterogeneity. Their development is regulated by signals provided by developing thymocytes. Human CD3ϵ transgenic mice (tgϵ26 mice), in which T-cell development is blocked at the early DN1 stage, have a disorganized cortex with cTECs arrested at the CD40^-^MHCII^lo^ stage (15, 16). However, the transplantation of tgϵ26 recipients with bone marrow cells from *Rag2*^-/-^ mice, exhibiting a subsequent block at the DN3 stage, restores the cortical organization (4, 17). Furthermore, cTECs with a CD40^+^MHCII^hi^ phenotype develop in the thymus of *Rag1*^-/-^ mice (16). Thus, cTEC development requires signals from thymocytes beyond the DN1 stage. Nevertheless, the cell-signaling pathways responsible for their development remain to be determined.

## Features of Medullary TECs

Compared to cTECs, mTECs are better characterized, likely because they are more abundant. mTECs have the unique ability to express up to 85%–90% of the genome and virtually all protein-coding genes (18). This promiscuous gene expression program is induced by the autoimmune regulator (Aire) and the transcription factor Fez family zinc finger 2 (Fezf2) (18, 19). mTECs contain two main subsets identified on MHCII and CD80 cell surface expression levels: MHCII^lo^CD80^lo^ (mTEC^lo^) and MHCII^hi^CD80^hi^ (mTEC^hi^) (20). These two subsets are heterogeneous based on distinct markers and functional properties. mTEC^lo^ contain mTEC^hi^ precursors expressing alpha-6 integrin (*Itga6*) and Sca1 (*Ly6a*) (21–23). They also comprise CCL21^+^ mTECs implicated in the migration of positively-selected thymocytes into the medulla (24). Cell fate mapping studies have identified that mTEC^lo^ contain post-Aire cells characterized by the loss of Aire protein and low surface levels of MHCII and CD80 molecules (25–27). Another subset of terminally differentiated mTECs closely resembling the gut chemosensory epithelial tuft cells are also present in mTEC^lo^ (28, 29). These cells express the doublecortin-like kinase 1 (Dclk1) marker and the transcription factor Pou2f3. Thus, the mTEC^lo^ compartment is particularly heterogeneous, containing not only mTEC^hi^ precursors but also CCL21^+^, post-Aire and tuft-like mTECs. The mTEC^hi^ compartment is also diverse, containing Aire^-^Fezf2^+^ and Aire^+^Fezf2^+^ subsets.

Single-cell transcriptomic analyses have identified dozens of TEC subsets, including perinatal cTECs, mature cTECs, mTEC progenitors, Aire^+^, post-Aire and tuft-like mTECs (12, 28, 30). Among them, two other minor subsets termed neuronal and structural TECs have been identified based on their expression signatures associated with neurotransmitters and extracellular matrix such as collagens and proteoglycans (12). Further investigations are required to define their anatomical localization and function. Interestingly, a subset of proliferating mTECs expresses substantial levels of *Aire*, suggesting that it corresponds to a maturational stage just before Aire^+^ mature mTECs (12, 30).

In humans, cTECs and mTECs are defined as EpCAM^int^CDR2^hi^ and EpCAM^hi^CDR2^-^, respectively (31). AIRE and FEZF2 are also expressed in human mTECs, indicating a conserved mechanism for the regulation of tissue-restricted self-antigens (19, 32, 33). Recent single-cell transcriptomic analyses across the lifespan showed a largely conserved TEC heterogeneity in humans (34). cTECs are more abundant during early fetal development, then a population with cTEC and mTEC properties appears in the late fetal and pediatric human thymus and lastly mTECs are dominants. Interestingly, two rare TEC subsets expressing *MYOD1* and *NEUROD1* genes that resemble myoid and neuroendocrine cells, respectively, were also identified. Although these subsets are preferentially located in the medulla, their respective function remains to be studied.

## RANK-RANKL Axis in mTEC Expansion and Differentiation

**Figure 1:**
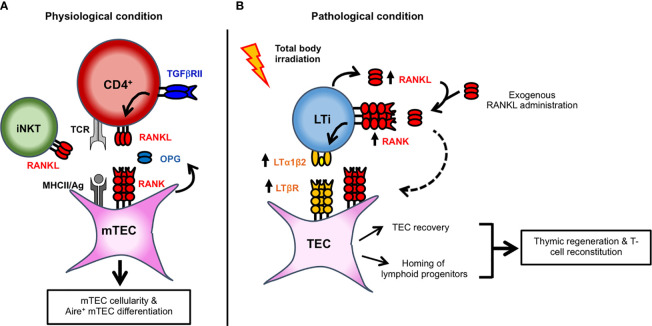
Key cellular actors implicated in RANK-RANKL signaling axis and in mTEC development and thymic regeneration. **(A)** TGFβRII signaling upregulates the expression of RANKL in autoreactive CD4^+^ thymocytes. RANKL, expressed by autoreactive CD4^+^ thymocytes and iNKT cells, controls mTEC cellularity and Aire^+^ mTEC differentiation. By binding to RANKL as a decoy receptor, OPG produced by mTECs inhibits RANK signaling and thereby regulates mTEC development. **(B)** Upon total body irradiation, radio-resistant LTi cells and CD4^+^ thymocytes upregulate RANKL expression. LTi cells also upregulate RANK receptor. RANKL upregulation or exogenous RANKL administration induces the heterocomplex LTα1β2 at the cell surface of LTi. RANKL could also stimulate RANK signaling in TECs (dashed arrow). TECs also upregulate the corresponding LTβR receptor after total body irradiation. In turn, LTα1β2-LTβR axis activation induces TEC regeneration by promoting their proliferation and survival. Furthermore, by inducing the expression of chemokines and adhesion molecules, this axis also favors the thymus homing of circulating T-cell progenitors.

The tumor necrosis factor receptor superfamily (TNFRSF) member, receptor activator of nuclear factor kappa-B (RANK; encoded by *Tnfrsf11a*) and its cognate ligand RANKL (encoded by *Tnfsf11*) play a privileged role in mTEC expansion and differentiation. During embryonic development, RANK gradually increases and is expressed by Aire^+^ mTEC precursors (35). In the adult, RANK is expressed by subsets that reside within mTEC^lo^ and mTEC^hi^, including CCL21^+^ and Aire^+^ cells (36). Importantly, the RANK-RANKL axis activates the classical and non-classical NF-κB signaling pathways that control the development of Aire^+^ mTECs (37). In the embryonic thymus of RANK- or RANKL-deficient mice, Aire^+^ mTECs are absent, indicating that this axis governs the emergence of Aire^+^ mTECs (37, 38). At this stage, RANKL is provided by CD4^+^CD3^-^ lymphoid tissue inducer (LTi) cells and invariant V*γ*5^+^ dendritic epidermal T cells (DETC) (39). Nevertheless, other hematopoietic cells might be implicated since few Aire^+^ mTECs are still detected in the embryonic thymus of mice lacking both LTi cells and DETC. In the postnatal thymus, the absence of RANK or RANKL leads to a partial reduction in Aire^+^ mTECs, showing that other signal(s) are involved in mTEC differentiation after birth (37, 40). Although *Cd40*^-/-^ and *Cd40lg^-^*^/-^ mice show subtle defects in Aire^+^ mTECs, these cells are further decreased in *Tnfrsf11a*^-/-^ × *Cd40^-^*^/-^ double-deficient mice compared to *Tnfrsf11a*^-/-^ mice, showing that RANK and CD40 cooperate to induce mTEC differentiation after birth (37). In the postnatal thymus, whereas CD40L is exclusively provided by CD4^+^ thymocytes, RANKL is higher in CD4^+^ than in CD8^+^ thymocytes and detected in iNKT cells (40–42) **(****Figure 1A****)**. The contribution of LTi, DETC and iNKT cells in the adult might be limited due to their paucity compared to the large numbers of CD4^+^ thymocytes. This assumption is corroborated by the fact that mice deficient in CD4^+^ thymocytes have a dramatic reduction in Aire^+^ mTECs and an underdeveloped medulla (41, 43).

RANKL is primarily synthesized as a membrane-bound trimeric complex that can be cleaved into its soluble form by proteases (44). A recent study showed that mice lacking soluble RANKL have normal numbers of Aire^+^ mTECs, indicating that membrane-bound rather than soluble RANKL induces their differentiation (45). Accordingly, RANKL and CD40L signals are delivered by CD4^+^ thymocytes in the context of antigen-specific TCR/MHCII-mediated interactions with mTECs (41, 43, 46). This is well illustrated in Rip-mOVAxOTII-*Rag2*^-/-^ mice, in which the Rip-mOVA transgene drives the expression of membrane-bound OVA in mTECs allowing high affinity interactions with OVA-specific OTII CD4^+^ thymocytes. Aire^+^ mTECs develop in these mice in contrast to OTII-*Rag2*^-/-^ mice. RANKL in CD4^+^ thymocytes is likely regulated by TGFβRII signaling (47). Mice lacking TGFβRII in αβ thymocytes at the early DP stage (*Cd4*-cre x *Tgfbr2*^fl/fl^ mice) have reduced RANKL levels in Helios^+^ autoreactive CD4^+^ thymocytes. Conversely, the stimulation of purified autoreactive CD4^+^ thymocytes with TGF-β increases RANKL expression. This upregulation is prevented by MAPK pathway inhibitors, indicating that TGFβRII signaling induces RANKL by its SMAD4/TRIM33-independent pathway. Similarly, TGF-β stimulation was shown to increase RANKL in TCR-activated T-cell hybridoma (48).

RANK signaling is regulated by the soluble decoy receptor for RANKL, osteoprotegerin (OPG; encoded by *Tnfrsf11b*), which inhibits RANKL interaction with its receptor RANK. OPG deficiency leads to an increased mTEC cellularity resulting in enlarged medulla with an enrichment in Aire^+^ mTECs (49). Mice harboring a *Tnfrsf11b* deletion in mTECs have increased numbers of total and Aire^+^ mTECs, similarly to *Tnfrsf11b^-/-^* mice (50). Thus, OPG produced locally by mTECs rather than serum OPG regulates mTEC cellularity and differentiation. RANK activates *Aire* expression by the NF-κB signaling because *Aire* contains in its upstream coding region a highly conserved noncoding sequence 1 (CNS1) with two NF-κB binding sites (51, 52). CNS1-deficient mice consequently lack *Aire* expression in mTECs and show many characteristics of *Aire^-^*^/-^ mice including reduced Aire-dependent tissue-restricted self-antigens. Noteworthy, the RANK-RANKL axis does not only induce *Aire* by itself but also controls mTEC cellularity and differentiation. In addition to Aire^+^ mTECs, *Tnfsf11^-/-^* mice show reduced numbers of mTEC^lo^ and mTEC^hi^ (37). Conversely, *Tnfrsf11b^-/-^* mice have increased numbers of CCL21^-^ and CCL21^+^ mTEC^lo^ and Aire^-^ and Aire^+^ mTEC^hi^ (36, 49). Accordingly, the stimulation of 2-deoxyguanosine-treated thymic lobes with RANKL show increased mTEC cellularity including Aire^+^ mTEC^hi^, which is further augmented by the addition of CD40L protein (43, 53). Furthermore, *in vivo* anti-RANKL blockade results in a severe depletion of around 80% of mTECs with a substantial loss of mTEC^lo^ and Aire^+^ mTEC^hi^ (49). In addition to control Aire^+^ mTECs, RANK signaling therefore regulates the overall mTEC cellularity.

In humans, scRNA-seq data indicate that RANK is expressed by Aire^+^ mTECs (34). Interestingly, the stimulation of primary human mTECs with RANKL leads to the upregulation of *AIRE* mRNA, suggesting a conserved role for RANK signaling (54). Given the implication of RANK-RANKL axis in bone resorption, a monoclonal antibody specific of human soluble and membrane-bound RANKL, Denosumab, has been developed to inhibit osteoclast development and activity. Denosumab is now used in therapy to treat osteoporosis, primary bone tumors and bone metastases (55, 56). Nevertheless, considering the importance of RANK-RANKL axis in Aire^+^ mTEC differentiation, it remains to be defined whether this treatment could affect central tolerance and increase the risk of autoimmunity.

## Sensibility of TECs to Myeloablative Conditioning Regimen

**Figure 2:**
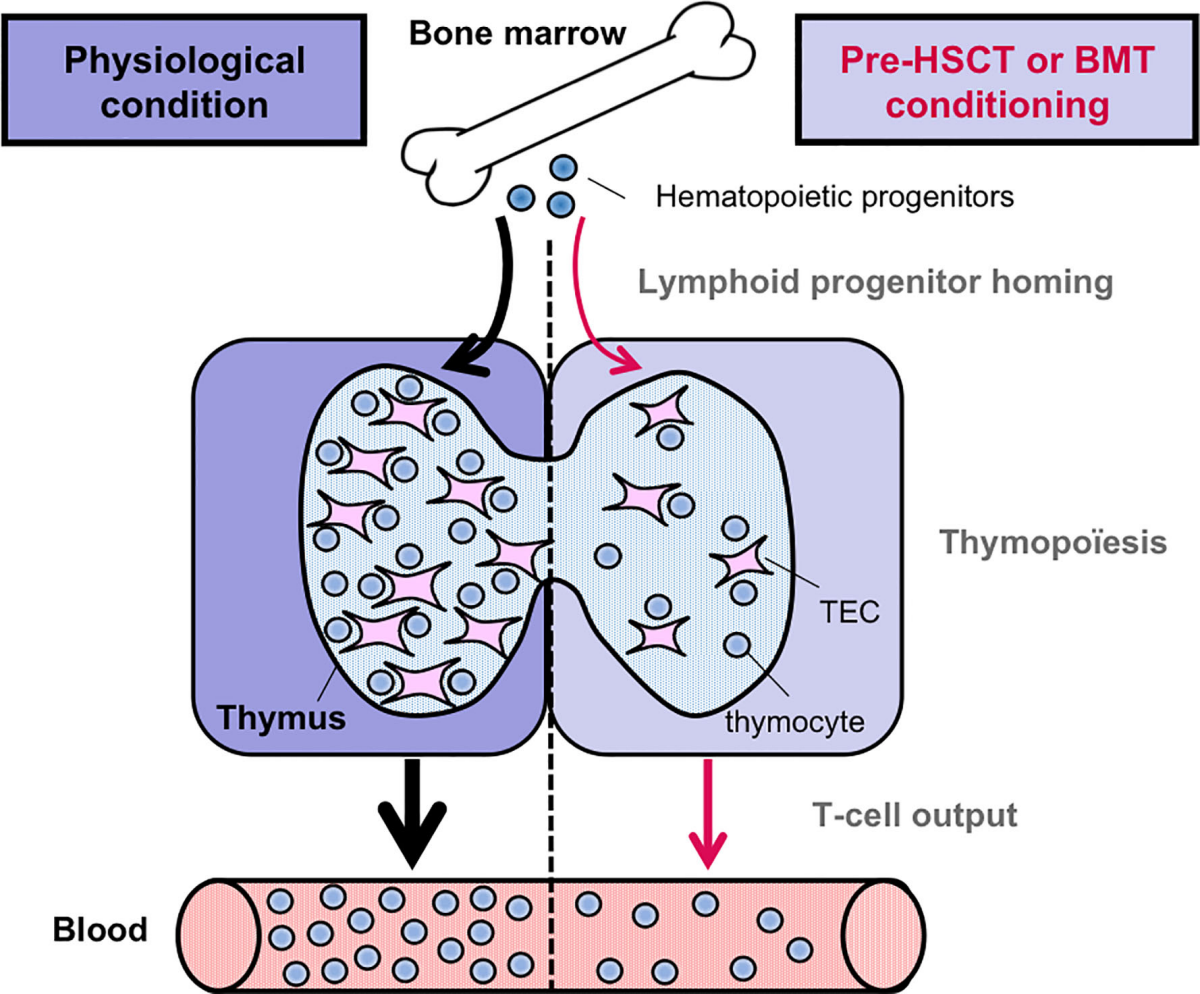
Pre-transplantation conditioning regimen alters thymic-dependent T-cell production. In contrast to the physiological condition, the homing ability of circulating T-cell progenitors is reduced after pre-HSCT or BMT conditioning regimen. Furthermore, T-cell production is also reduced, notably due to TEC damages induced by myeloablative regimens. Consequently, the output of newly generated naïve T cells is diminished.

Myeloablative treatments such as radiation and chemotherapy deplete hematopoietic cells and in particular DP thymocytes that are extremely sensitive. These treatments also impair the recruitment of circulating T-cell progenitors and induce damages on TECs **(****Figure 2****)**. Consequently, the generation of newly produced naïve T cells is reduced. Since TECs dictate the size of stromal niches, TEC injury contributes in a delayed T-cell reconstitution upon bone marrow (BMT) or hematopoietic stem transplantation (HSCT). In humans, allogeneic HSCT survivors are immunodeficient in T cells for at least 1 year, a period of high susceptibility to opportunistic infections, autoimmunity or tumor relapse, increasing the risk of morbidity and mortality (57, 58). Although innate cells and antibodies may limit viral infections, cytotoxic CD8^+^ T cells and helper CD4^+^ T cells are essential in viral clearance and the prevention of recurrent infections. T-cell recovery thus protects from lethality after BMT or HSCT. Importantly, T-cell immunity relies on the regeneration of the thymus and its capacity to produce naïve T cells. Total body irradiation (TBI) leads rapidly in a profound reduction of the cortex due to the loss of DP thymocytes and a substantial decrease of the medulla (59). Both cTECs and mTECs are radiosensitive (60, 61). Among mTECs, Aire^+^ mature mTECs are lost upon TBI and treatment with the chemotherapy agent cyclophosphamide or the immunosuppressant cyclosporine A, used to prevent allograft rejection (61, 62). However, the effects of such treatments on the recently identified dozens of TEC subsets remain to be investigated. Remarkably, the injured thymic tissue retains potent regenerative capacity. Targeting the pathways implicated in endogenous TEC regeneration is expected to improve thymic-dependent T-cell recovery. Potential strategies based on keratinocyte growth factor (KGF), IL-22 or Bone Morphogenic Protein 4 (BMP4) have been reviewed in (58, 63, 64). Strategies based on FOXN1 protein or cDNA administration also improve TEC regeneration both in the context of HSCT and aging (65, 66). A novel role for the RANK-RANKL axis in TEC regeneration and T-cell recovery is highlighted below.

## RANK-RANKL Axis in TEC Regeneration

RANKL is upregulated in radio-resistant LTi cells and CD4^+^ thymocytes during the early phase of thymic regeneration after total body irradiation (TBI) (61, 67, 68). Although LTi cells are rare in the thymus, they express a higher level of RANKL than CD4^+^ thymocytes after TBI (61). Interestingly, the administration of a neutralizing anti-RANKL antibody impairs TEC regeneration, emphasizing an important role for RANKL in endogenous TEC recovery. Conversely, RANKL protein administration increases TEC numbers at a level close to unirradiated mice. RANKL enhances cTEC and mTEC numbers, including Aire^+^ mTEC^hi^ and TEPC-enriched cells, likely by stimulating their proliferation and survival. These observations are in agreement with a previous study indicating that RANKL increases *in vitro* the proliferation of cortical and medullary TEC cell lines (69). Of clinical relevance, RANKL administration upon BMT boosts not only the regeneration of several TEC subsets but also increases T-cell progenitor homing **(****Figure 1B****)** (61). This latter effect could be explained by an enhanced cellularity of endothelial cells upon RANKL administration although further investigations are required. Consequently, this treatment ameliorates *de novo* thymopoiesis and peripheral T-cell reconstitution. Noteworthy, a single course of RANKL after BMT boosts thymic regeneration at least during 2 months, indicative of a lasting effect. This therapeutic strategy is also efficient in aged individuals in whom T-cell recovery upon BMT is less efficient and delayed (70). Age-related thymic involution results in a disrupted thymic architecture with a reduced TEC cellularity, which alters T-cell production (71). RANKL treatment could be thus of special interest to the elderly, although further studies are required.

Mechanistically, RANKL upregulates another TNF family ligand, lymphotoxin α (LTα; encoded by *Lta*), expressed as a membrane anchored LTα1β2 heterocomplex, in LTi of recipient origin **(****Figure 1B****)** (61). Conversely, the RANK-Fc antagonist fully blocks LTα1β2 upregulation. Noteworthy RANKL also induces LTα1β2 expression in LTi cells during lymph node formation (72). Likewise RANKL, LTα is upregulated during the early phase of thymic regeneration. Since CD4^+^ thymocytes upregulate RANKL and since LTi cells express both RANK and its ligand, RANK signaling may be triggered in LTi in an autocrine and paracrine manner. Given that LTi cells upregulate RANKL, LTα1β2, IL-22, IL-23R, and ROR*γ*t after thymic injury (61, 68), these cells are likely in a quiescent stage at steady state and activated after irradiation to repair the injured thymic tissue. Accordingly, the depletion of ILC3, comprising LTi cells, in an experimental model of graft-versus-host disease (GVHD) results in impaired thymic regeneration (73). Interestingly, LTβR is also upregulated in cTECs, mTECs, and TEPC-enriched cells after TBI, suggesting that the LTα1β2-LTβR axis is implicated in TEC regeneration (61). At steady state, *Lta*^-/-^ mice show normal numbers of TEC subsets. In contrast, cTECs, mTECs including Aire^+^ mTEC^hi^ and TEPC-enriched cells are substantially reduced in these mice upon BMT. These observations indicate that the mechanisms implicated in TEC regeneration are distinct from those used at steady state. Furthermore, these mice show reduced numbers of early T-cell progenitors (ETPs) because LTα controls the homing capacity of circulating T-cell progenitors by regulating the expression of CCL19 and CCL21 in TECs and ICAM-1, VCAM-1, and P-selectin in endothelial cells, all implicated in T-cell progenitor homing (61, 74). Similarly, *Ltbr*^-/-^ mice have an altered recruitment of T-cell progenitors after sublethal TBI (75). In agreement with defective TEC regeneration and T-cell progenitor homing, BM-transplanted *Lta*^-/-^ mice have impaired thymic and peripheral T-cell reconstitution. These beneficial effects induced by RANKL depend on LTα since they are essentially lost when RANKL is administered in *Lta*^-/-^ recipients. RANKL administration thus constitutes a novel therapeutic strategy to improve T-cell function recovery after thymic injury. Interestingly, RANK and LTβR expression is conserved in the human thymus, opening potential therapeutic perspectives (34). Besides applications linked to myeloablative conditioning regimen, these *in vivo* findings open new avenues to treat patients whose thymus has been severely damaged by aging, viral infections, or malnutrition.

## Author Contributions

The author confirms being the sole contributor of this work and has approved it for publication.

## Funding

This work received funding from the Agence Nationale de la Recherche (grant ANR-19-CE18-0021-01, *RANKLthym* to MI) and was supported by institutional grants from Institut National de la Santé et de la Recherche Médicale, Centre National de la Recherche Scientifique and Aix-Marseille Université.

## Conflict of Interest

The author declares that the research was conducted in the absence of any commercial or financial relationships that could be construed as a potential conflict of interest.
